# Socioeconomic inequalities in food outlet access through an online food delivery service in England: A cross-sectional descriptive analysis

**DOI:** 10.1016/j.apgeog.2021.102498

**Published:** 2021-08

**Authors:** Matthew Keeble, Jean Adams, Tom R.P. Bishop, Thomas Burgoine

**Affiliations:** a UKCRC Centre for Diet and Activity Research (CEDAR), MRC Epidemiology Unit, University of Cambridge School of Clinical Medicine, Box 285 Institute of Metabolic Science, Cambridge Biomedical Campus, Cambridge, CB2 0QQ, UK; b MRC Epidemiology Unit, University of Cambridge School of Clinical Medicine, Box 285 Institute of Metabolic Science, Cambridge Biomedical Campus, Cambridge, CB2 0QQ, UK

**Keywords:** Fast foods, Food environment, Food outlet access, GIS, Online food delivery services, Public health

## Abstract

Online food delivery services facilitate ‘online’ access to food outlets selling food prepared away-from-home. Online food outlet access has not previously been investigated in England or across an entire country. Systematic differences in online food outlet access could exacerbate existing health inequalities, which is a public health concern. However, this is not known. Across postcode districts in England (n = 2118), we identified and described the number of food outlets and unique cuisine types accessible online from the market leader (Just Eat). We investigated associations with area-level deprivation using adjusted negative binomial regression models. We also compared the number of food outlets accessible online with the number physically accessible in the neighbourhood (1600m Euclidean buffers of postcode district geographic centroids) and investigated associations with deprivation using an adjusted general linear model. For each outcome, we predicted means and 95% confidence intervals. In November 2019, 29,232 food outlets were registered to accept orders online. Overall, the median number of food outlets accessible online per postcode district was 63.5 (IQR; 16.0–156.0). For the number of food outlets accessible online as a percentage of the number accessible within the neighbourhood, the median was 63.4% (IQR; 35.6–96.5). Analysis using negative binomial regression showed that online food outlet access was highest in the most deprived postcode districts (n = 106.1; 95% CI: 91.9, 120.3). The number of food outlets accessible online as a percentage of those accessible within the neighbourhood was highest in the least deprived postcode districts (n = 86.2%; 95% CI: 78.6, 93.7). In England, online food outlet access is socioeconomically patterned. Further research is required to understand how online food outlet access is related to using online food delivery services.

## Highlights

• Online food delivery services provide access to food prepared away-from-home.
• We describe ‘online’ access to outlets selling this food across England.
• Around one-third of food outlets were registered to accept orders online.
• Online food outlet access increases existing neighbourhood food outlet access.
• Online food outlet access was greatest in the most deprived areas.

## 1 Introduction

In 2018, half of food expenditure in the USA was on food prepared away-from-home (United States Department of Agriculture Economic Research Service, 2020), and between 2008 and 2012 over one quarter of adults in the UK consumed at least one meal prepared away-from-home each week (Adams et al., 2015). Food available away-from-home is often characterised by high levels of energy, fat and salt, and on the whole, is less healthy than food prepared at home (Jaworowska et al., 2012; Jaworowska et al., 2014; Robinson et al., 2021). Decisions related to when and where food is purchased are multifactorial (Rutter, 2012; Turner et al., 2018), however, the built environment has a recognised influence on food purchasing practices, and the consumption of food prepared away-from-home (Lam et al., 2021). The number of physically accessible food outlets is a geographical dimension of “access”, where outlets are suggested to act as an environmental cue that results in their use (Caspi et al., 2012; Penchansky & Thomas, 1981). Accordingly, environments providing abundant access to food outlets selling unhealthy food prepared away-from-home have been conceptualised as obesogenic (Lake & Townshend, 2006).

Evidence from a growing body of cross-sectional and longitudinal research investigating neighbourhood food outlet access now exists (Eckert & Vojnovic, 2017; Helbich et al., 2017). The evidence base as a whole regarding associations between food outlet access with food related practices and related outcomes is equivocal (Fleischhacker et al., 2011; Wellard-Cole et al., 2021). In part, this is a reflection of methodological heterogeneity across studies, including the use of different geographical measures of food outlet access and conceptualisations of neighbourhood food environments, as well as varying food environment contexts across countries (Wilkins et al., 2017; Wilkins et al., 2019). Nonetheless, in two UK studies that used similar methods and were conducted in large samples of adults, neighbourhood exposure to fast-food outlets was positively associated with fast-food consumption (Burgoine et al., 2014; Burgoine et al., 2016), and this food practice has been associated with excess weight gain over time (Pereira et al., 2005). Moreover, it has been consistently reported across international contexts that more deprived areas have a higher number of food outlets selling food prepared away-from-home (Block et al., 2004; Maguire et al., 2015; Simon et al., 2008; Smoyer-Tomic et al., 2008), which may be contributing to observed inequalities in diet and health. Importantly, however, the ability to acquire food prepared away-from-home is no longer restricted to physical food outlet access, and a notable limitation of previous research is that alternative ways of accessing this food were not considered, which could prove to be important.

Online food ordering and delivery services, more commonly known (and referred to hereafter) as online food delivery services, facilitate online food outlet access and have grown in popularity. In 2020, a number of prominent online food delivery services were in operation internationally. *Deliveroo* was available in 12 countries (Deliveroo, 2019), whilst *Grubhub* was available in over 4000 cities across the USA (Grubhub, 2020). However, *Just Eat* *Takeaway.com* (including its subsidiaries) was available in 23 countries (Just Eat, 2020), and was the market leader in the UK with regards to the number of food outlets registered to accept orders (around 30,000) and the annual number of orders processed (almost 170 million) (Just Eat, 2021b; Statista., 2020).

Unlike visiting food outlets in person, online food delivery service customers use internet-enabled devices to visit platforms that facilitate ‘online’ access to food outlets registered to accept orders (Allen et al., 2017). Based on the customer's location, they receive information about all food outlets that they could order from (i.e. those that will deliver to them). Customers select a food outlet and place their order through the online food delivery service platform. Orders are then forwarded to the outlet where meals are prepared. When ready, meals are delivered by couriers who work for either the online food delivery service or the food outlet.

As with physical food outlet access, where a greater number of food outlets leads to a greater number of opportunities to purchase unhealthy food prepared away-from-home (Egger & Swinburn, 1997; Swinburn et al., 2011), it is possible that greater online food outlet access leads to a greater number of purchasing opportunities and is positively associated with online food delivery service use. As described, there are known social inequalities in access to food outlets selling food prepared away-from-home in England, with outlets selling this food more prevalent in more deprived neighbourhoods (Maguire et al., 2015). Similar trends have also been described elsewhere (Nijman & Wei, 2020). Whilst orders placed through online food delivery services are made through online platforms, food sold is typically prepared in the kitchens of food outlets that exist in or near the customer's neighbourhood, which facilitates rapid and efficient delivery (Allen et al., 2017). As such, inequalities in physical food outlet access may be reflected in online food outlet access. Since foods sold through online platforms are recognised as unhealthy, with an energy-dense and nutrient-poor composition (Wang et al., 2021), existing systematic differences in diet and diet-related health could be exacerbated. However, this remains poorly investigated and unconfirmed. We are aware of no work from the UK, and research completed elsewhere has focussed on small geographical areas within a limited number of cities (Partridge et al., 2020; Poelman et al., 2020). Therefore, the full extent of nationwide variation in online food outlet access and potential differences across the full socioeconomic gradient that might only be observed across an entire country, remain unknown. This variation is important to understand since vulnerable sociodemographic groups may be disproportionately affected by greater food outlet access that exists across multiple modes of order.

Beyond the number of accessible food outlets, other factors could also influence online food delivery service use. Broadly speaking, customers select food outlets based on the cuisine they sell (Caspi et al., 2012; Furst et al., 1996). Within the context of online food delivery services, access to a greater number of unique cuisine types could mean that customers can access food prepared away-from-home regardless of the cuisine type they desire, resulting in greater use. To our knowledge, access to different cuisine types has not been addressed in the limited number of existing studies on online food delivery services.

### 1.1 Study aims

In this cross-sectional, area-based study, we aimed to: describe access to food outlets and unique cuisine types through an online food delivery service across England; compare online food outlet access with physical food outlet access within the neighbourhood; and examine whether and to what extent these measures were associated with deprivation.

## 2 Materials and methods

### 2.1 Study setting

The setting for our study was England and we completed analyses at the postcode district level. This analytic scale reflects how food outlets registered to accept orders through Just Eat delineate their ‘delivery area’ (see section 2.3.1). Postcode districts are found within the first half of a full postcode, which is formally known as the outward code (Beacon Dodsworth, 2020). For example, the *postcode district* of the *postcode* “CB2 0QQ” is “CB2”. We used boundary data from 2012, provided by the UK data service (UK Data Service, 2020), to map postcode districts in England in a geographic information system (GIS) (ArcGIS version 10.7.1; ESRI Inc., Redlands, CA). We included 2118 postcode districts in England, including those entirely within, as well as those whose boundary intersected the English border. Whilst postcode districts vary in size, based on data from the 2011 census, the median postcode district population was 23,610 (IQR; 13,320–34,560).

### 2.2 Exposure: relative deprivation

We used the 2019 Index of Multiple Deprivation (IMD) to measure relative deprivation. This is a compound measure including metrics across seven domains: income deprivation, employment deprivation, crime, health deprivation and disability, education, skills and training deprivation, barriers to housing and services and living environment deprivation (Ministry of Housing Communities and Local Government, 2019). Relative deprivation scores are available for lower super output areas (LSOAs) in England, which are administrative boundaries with a mean residential population of 1500 people (Office for National Statistics, 2016b). As LSOAs are typically geographically smaller than postcode districts, we calculated the average IMD score of LSOAs within and intersecting the boundary of each postcode district (Ministry of Housing Communities and Local Government, 2019). For analyses, we split postcode districts into deciles based on IMD score, with decile 1 containing the least deprived areas.

### 2.3 Outcomes

Table 1 summarises each of the outcome measures we used in our study.

**Table 1:** Summary of outcome measures and exposure-outcome relationships investigated.

Exposure	Outcome	Outcome description	Geography	Covariates added to controlled model
Postcode district relative deprivation, modelled as deciles (D): D1 = least deprived postcode districts.	Percentage of food outlets registered to accept orders online	The number of food outlets registered to accept orders online, expressed as a percentage of the number of food outlets within a postcode district (bounded, 0–100%).	Postcode district	Postcode district rural urban classification
				Postcode district population density: usual residential and usual workday
	Number of food outlets accessible online	The number of food outlets accessible online based on a postcode district being listed in the delivery area of a food outlet registered to accept orders online.	Postcode district	Postcode district rural urban classification
				Number of food outlets in postcode district
				Postcode district population density: usual residential and usual workday
	Number of unique cuisine types accessible online	The number of unique cuisine types accessible online from food outlets registered to accept orders online who included a postcode district in their delivery area.	Postcode district	Postcode district rural urban classification
				Number of food outlets in postcode district
				Postcode district population density: usual residential and usual workday
				Number of food outlets accessible online
	Percentage of neighbourhood food outlets accessible online	The number of food outlets accessible online expressed as a percentage of the number physically accessible within the neighbourhood (unbounded, may exceed 100%).	1600m Euclidean radius ‘neighbourhood’ buffer of postcode district geographic centroid	Postcode district rural urban classification
				Postcode district population density: usual residential and usual workday

#### 2.3.1 Number of food outlets and unique cuisine types accessible online

Whilst several online food delivery services are available in England, information about all food outlets registered to accept orders through the market leader (Just Eat), including their opening hours, menus, delivery fees and customer reviews, is publicly available. We completed pilot work for one postcode district in England, and found that 95% of food outlets registered to accept orders through a competitor (Deliveroo) were also registered to accept orders through Just Eat (see appendix). Moreover, unlike competitors food outlets registered to accept orders through Just Eat are reported to be accessible across England (Just Eat, 2021a). As such, we used data from Just Eat as a proxy for online food outlet access. Given that the aims of our study were related to online food delivery services generally, we refer to Just Eat as the ‘online food delivery service’ hereafter.

In November 2019, we used a web-browser extension to collect data about food outlets accessible online in England, Wales and Scotland (Webscraper, 2019). First, on one weekday, we identified all food outlets registered to accept orders. Second, within 72-h, we visited the profile of each outlet on the online food delivery service website and collected information on their physical location, the types of cuisine sold, and their delivery area, which is a list of all postcode districts to which they delivered. Based on their postcode, we geocoded food outlets registered to accept orders through the online food delivery service using GeoConvert, which is maintained by the UK Data Service (GeoConvert, 2020). When geocoding was not successful we used Doogal, which is a free web-based resource (Doogal, 2020). We were unable to geocode seven food outlets (0.02%). We identified and geocoded 29,232 food outlets within postcode districts in England, and mapped them in our GIS using supplied coordinates.

We used the number of food outlets and the number of unique cuisine types that were accessible online, through the online food delivery service, as our outcome measures. To identify the number of accessible food outlets, we counted the number of food outlets registered to accept orders through the online food delivery service that listed each postcode district in their delivery area. To identify the number of accessible unique cuisine types, we counted the number of different cuisines listed by the accessible food outlets. Food outlet owners can select multiple unique cuisine types to describe their food outlet. As a result, the number of unique cuisine types accessible online could be greater than the number of food outlets.

#### 2.3.2 Percentage of food outlets registered to accept orders online

We used data from Ordnance Survey's Points of Interest (OS POI) dataset, which is commercial data that contains information about food outlets from over 170 suppliers (Ordnance Survey, 2020), and is one of the most complete sources of food outlet location data available for England (Burgoine & Harrison, 2013). Data from OS POI has been used in previous research investigating physical food outlet exposure (Penney et al., 2018). We used data from June 2019 and extracted information for the following food outlet categories: “*Fast food and takeaway outlets*” (food outlets selling food for consumption away-from-the premises), “*Fast food delivery services*” (food outlets selling food for delivery, not explicitly through online services), “*Fish and Chip shops”* (food outlets selling a traditional British cuisine typically for consumption away-from-the premises) and “*Restaurants*” (food outlets selling food for consumption inside the premises) (PointX, 2006). We selected these categories based on *a priori* knowledge that they included food outlets typically registered to accept orders through online food delivery services. We mapped the locations of food outlets within each postcode district using coordinates supplied in OS POI data, which have a stated accuracy of 1m (Ordnance Survey, 2019).

We calculated the percentage of food outlets physically located within each postcode district that were registered to accept orders through the online food delivery service. To calculate this measure we compared the number of food outlets located within each postcode district that were registered with the online food delivery service, with the number of food outlets within each postcode district listed in OS POI data. The number of food outlets registered to accept orders through the online food delivery service should not exceed the number of food outlets located within each postcode district, therefore, we used a bounded measure (between 0 and 100%). However, we did not identify and match individual food outlets listed in both datasets.

#### 2.3.3 Percentage of neighbourhood food outlets accessible online

We also expressed the number of food outlets accessible online as a percentage of the number listed in OS POI data that were physically accessible within the neighbourhood food environment. We defined the neighbourhood food environment as a 1600m (1 mile) Euclidean (straight-line) distance from the geographic centre of each postcode district. Physical exposure to food outlets within this distance has been associated with dietary and shopping behaviours (Smith et al., 2010), and has previously been used to conceptualise neighbourhood food environments (Wilkins et al., 2019). The number of food outlets accessible online may exceed the number physically accessible within the neighbourhood, therefore, this percentage could be greater than 100%.

### 2.4 Covariates

Food sold through online food delivery services is typically prepared in existing food outlets in the neighbourhood, and outlets selling food prepared away-from-home tend to concentrate in urban areas, reflecting greater demand resulting from higher population densities (Needham et al., 2020). Therefore, online food outlet access might be a function of physical food outlet access in the neighbourhood. We counted the number of food outlets in the neighbourhood using the four categories from OS POI data described in section 2.3.2., and included this as a covariate. Additionally, the number of unique cuisine types accessible online was positively related to the number of food outlets accessible online. Therefore, we used the number of food outlets accessible through the online food delivery service as a covariate when it was not part of the outcome measure. We used the 2011 rural urban classification (Office for National Statistics, 2016a), to categorise postcode districts as: ‘rural’ when LSOAs within or intersecting their boundary were most were most frequently rural (populations less than 10,000 people within combined settlements); ‘urban’ when LSOAs were most frequently urban (populations greater than 10,000 people within combined settlements); or ‘balanced’ when the number of rural and urban LSOAs was equal. We also included two measures of population density from the 2011 UK census: residential and workday population (Office for National Statistics, 2013, 2014). These measures reflect the number of individuals, including students and schoolchildren not living away from home during term-time, that usually reside in a postcode district (i.e. ‘usual residential population’), and the number of individuals usually working in a postcode district on a given day, regardless of their usual place of residence, plus usual residents who are unemployed (i.e. ‘usual workday population’), respectively. These data were available for 2088 (95.4%) postcode districts.

### 2.5 Statistical analyses

We used Stata version 16.1 (StataCorp LLC., College Station, TX, USA), to complete statistical analyses, with a significance threshold of p < 0.05 throughout. Table 1 outlines the exposure-outcome relationships we investigated and the covariates included in each controlled model. For each exposure-outcome relationship investigated, we included postcode districts with complete data on all relevant variables.

Data on the number of food outlets and unique cuisine types accessible online were not normally distributed and were over-dispersed. Therefore, we used negative binomial regression to investigate associations with postcode district deprivation. Negative binomial regression reports incidence rate ratios (IRRs) and 95% confidence intervals (CIs). In the context of our study, IRRs are the expected change in the outcome measure at each level of deprivation compared to the least deprived (decile 1). For both outcomes, we controlled for the number of food outlets within a postcode district, and postcode district rural urban classification and population density. When the number of unique cuisine types accessible online was the outcome, we also controlled for the number of food outlets accessible online.Where the outcome was the percentage of food outlets registered to accept orders online or the percentage of food outlets within the neighbourhood accessible online, we used general linear models to investigate associations with postcode district deprivation. For these outcomes, model coefficients are the difference in the percentage at each level of deprivation compared to the least deprived. For both of these outcomes, we controlled for postcode district rural urban classification and population density.

To aid interpretation of outcomes, we primarily report predicted means and 95% CIs calculated from IRRs or coefficients from models that controlled for covariates. We present these IRRs and coefficients in the appendix (Table A.2, Table A.3, Table A.4, Table A.5).

### 2.6 Sensitivity analyses

To test the sensitivity of our findings to the selection of four food outlet categories from OS POI data (“*Fast food and takeaway outlets*”, “*Fast food delivery services*”, “*Fish and Chip shops*”, “*Restaurants*”), we ran models that included the number of food outlets from five additional categories: “*Cafes, snack bars and tea rooms*”, “*Convenience stores*”, “*Supermarkets*”, “*Bakeries*”, “*Delicatessens*”. We present the findings from our sensitivity analyses in the appendix Table A.6, Table A.7, Table A.8, Table A.9.

## 3 Results

In November 2019, 29,232 food outlets across England were registered to accept orders through the online food delivery service (for total numbers see appendix: Table A.1).

### 3.1 Food outlet access across England

**Fig. 1:**
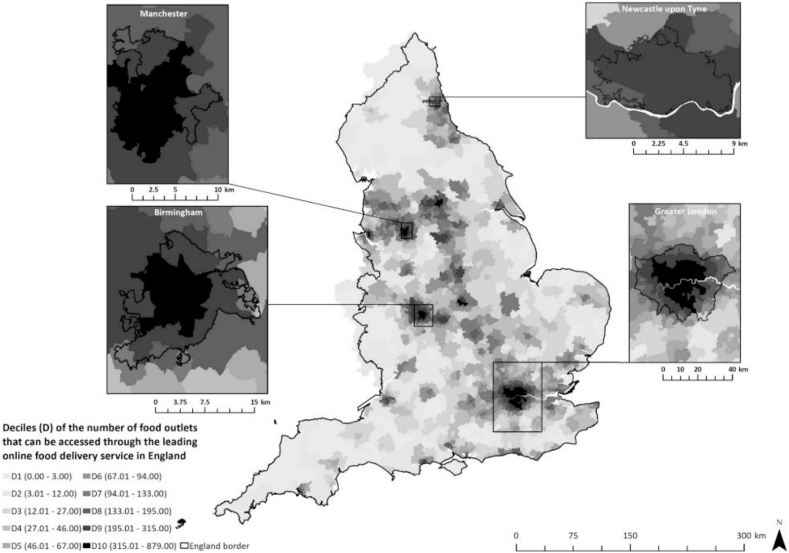
Deciles of the number (count) of food outlets accessible online across postcode districts in England (n = 2118), in November 2019.

Descriptive statistics summarising online and physical access to food outlets that predominantly serve food prepared away-from-home are shown in Table 2. Overall, the median number of food outlets physically located within postcode districts was 30.0 (IQR; 14.0–52.0). The median number of food outlets registered to accept orders online and located within postcode districts as a percentage of food outlets within postcode districts was 30.0% (IQR; 10.0–40.0). The median number of food outlets accessible online per postcode district was 63.5 (IQR; 16.0–156.0). Online access to food outlets was widespread, but varied, across England (Fig. 1). We observed clusters of postcode districts in the North East, North West, West-Midlands, and Greater London regions of England with a high number of food outlets accessible online. Postcode districts in these regions are typically urban and highly populated. From food outlets accessible online, the median number of unique cuisine types available was 39.0 (IQR; 16.0–68.0). When the number of food outlets accessible online was expressed as a percentage of the number physically accessible within the neighbourhood, the median was 63.4% (IQR; 35.6–96.5). The percentage of food outlets within a postcode district registered to accept orders online was greatest in postcode districts in decile 10 of deprivation (n = 50.0%: IQR; 40.0–60.0). Postcode districts in decile 10 of deprivation also had the greatest median online food outlet access (n = 186.0: IQR; 102.0–294.0), and the greatest median number of food outlets accessible online as a percentage of the number physically accessible within the neighbourhood (n = 77.4%: IQR; 62.2–107.7).

**Table 2:** Summary of measures for the online food delivery service and the physical food environment across postcode districts in England (n = 2118), stratified by deprivation.

	Deprivation decile^a^										
	1	2	3	4	5	6	7	8	9	10	All
Measure	(4.28–10.21) n = 214	(10.22–12.08) n = 210	(12.09–14.00) n = 213	(14.01–15.91) n = 211	(15.92–18.18) n = 211	(18.19–20.60) n = 212	(20.61–23.54) n = 212	(23.55–27.06) n = 212	(27.07–32.89) n = 212	(32.90–69.51) n = 211	n = 2118
Online food delivery service											
Food outlets registered^b^ (count)	3.0 (1.0–7.0)	3.0 (0.0–10.0)	3.0 (1.0–9.0)	5.0 (1.0–13.0)	5.0 (0.0–14.0)	6.0 (1.0–18.0)	9.5 (1.0–23.5)	14.5 (5.0–26.0)	21.5 (10.0–37.5)	24.0 (14.0–37.0)	7.0 (1.0–21.0)
Accessible food outlets (count)	41.0 (18.0–68.0)	31.0 (12.0–74.0)	30.0 (11.0–89.0)	44.0 (11.0–101.0)	41.0 (8.0–106.0)	62.0 (9.0–121.0)	76.0 (4.0–176.5)	92.5 (30.5–208.5)	143.0 (80.0–247.0)	186.0 (102.0–294.0)	63.5 (16.0–156.0)
Unique cuisine types accessible (count)	31.0 (18.0–43.0)	26.5 (13.0–47.0)	27.0 (12.0–47.0)	32.0 (13.0–55.0)	29.0 (10.0–54.0)	36.0 (10.0–59.5)	44.0 (6.5–72.0)	49.0 (24.0–83.5)	59.5 (40.5–84.0)	71.0 (48.0–95.0)	39.0 (16.0–68.0)
Physical food environment											
Food outlets within postcode^c^ district (count)	18.0 (9.0–31.0)	21.0 (10.0–38.0)	24.0 (10.0–39.0)	25.0 (11.0–45.0)	24.0 (12.0–43.0)	28.0 (11.0–52.5)	34.5 (18.5–56.5)	41.5 (21.5–68.0)	50.5 (29.5–81.0)	50.0 (29.0–76.0)	30.0 (14.0–52.0)
Food outlets within neighbourhood^c^ (count)	45.0 (26.0–93.0)	52.5 (24.0–97.0)	55.0 (26.0–129.0)	67.0 (29.0–142.0)	75.0 (29.0–154.0)	90.5 (31.0–169.0)	112.0 (34.0–223.5)	123.5 (49.0–274.5)	191.0 (105.5–290.5)	212.0 (146.0–343.0)	90.0 (36.0–200.0)
Percentage registered^d^ (%)	20.0 (10.0–30.0)	20.0 (0.0–30.0)	10.0 (0.0–30.0)	20.0 (10.0–40.0)	20.0 (0.0–40.0)	20.0 (10.0–40.0)	30.0 (0.0–50.0)	40.0 (20.0–50.0)	40.0 (30.0–50.0)	50.0 (40.0–60.0)	30.0 (10.0–40.0)
Percentage accessible online^e^ (%)	70.9 (40.4–115.1)	59.4 (33.3–100.0)	49.7 (27.3–91.3)	59.2 (29.2–96.4)	53.3 (26.3–97.1)	56.8 (28.3–88.4)	52.7 (23.1–82.4)	67.3 (40.1–91.9)	72.1 (52.6–95.0)	77.4 (62.2–107.7)	63.4 (35.6–96.5)

a Decile 1 = least deprived, decile 10 = most deprived. Data reported as median (IQR) unless stated.

b ‘Registered’ = registered to accept orders online, through the online food delivery service.

c Food outlet categories included: Fast food and takeaway outlets; Fast food delivery services; Fish and Chip shops; Restaurants. ‘Neighbourhood’ = 1600m Euclidean radius ‘neighbourhood’ buffer of postcode district geographic centroid.

d The number of food outlets registered to accept orders online as a percentage of the number of food outlets within a postcode district.

e The number of food outlets accessible online as a percentage of the number physically accessible within the neighbourhood.

### 3.2 Association between deprivation and the percentage of food outlets registered to accept orders online

**Fig. 2:**
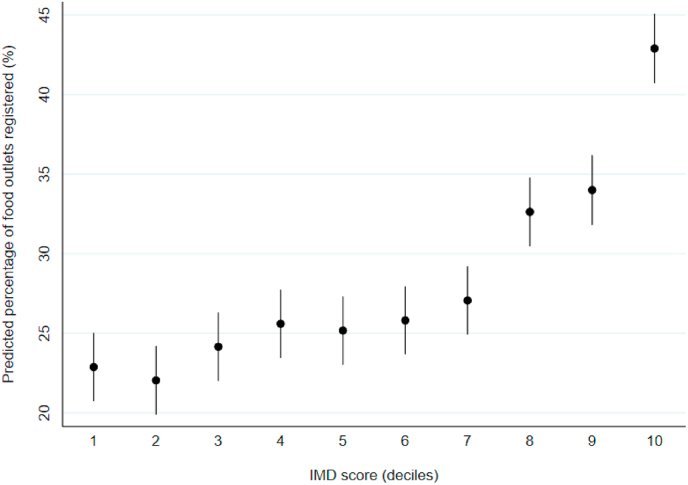
Percentage of food outlets within a postcode district registered to accept orders online, across postcode districts in England (n = 2084). Data points are predicted means with 95% CIs, calculated from coefficients estimated using a general linear model, controlled for postcode district rural urban classification and population density.

In our controlled model, we observed evidence suggestive of a positive dose-response association between the percentage of food outlets located within postcode districts registered to accept orders online and deprivation. Predicted means with 95% CIs, calculated from coefficients of our controlled model are shown in Fig. 2. Districts in deciles 8–10 had significantly greater percentages of food outlets registered to accept orders online than those in decile 1 (least deprived). In the most deprived postcode districts, 42.9% (95% CI: 40.7, 45.1) of food outlets were predicted to be registered to accept orders online, compared to 22.8% (95% CI: 20.7, 25.0) in the least deprived.

### 3.3 Association between deprivation and online food outlet access

**Fig. 3:**
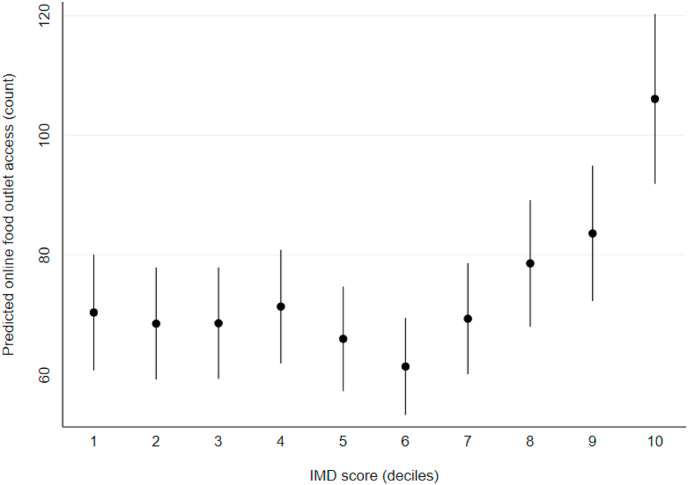
Number of food outlets accessible online across postcode districts in England (n = 2088). Data points are predicted means with 95% CIs, calculated from IRRs estimated using negative binomial regression, controlled for postcode district rural urban classification, population density, and the number of food outlets within the postcode district.

In our controlled model, there was limited evidence of a trend in online food outlet access across deprivation deciles (Fig. 3). However, the most deprived postcode districts (decile 10) had significantly greater online food outlet access (106.1 outlets; 95% CI: 91.9, 120.3), compared to the least deprived postcode districts (70.4 outlets; 95% CI: 60.8, 80.1).

### 3.4 Association between deprivation and unique cuisine type access

**Fig. 4:**
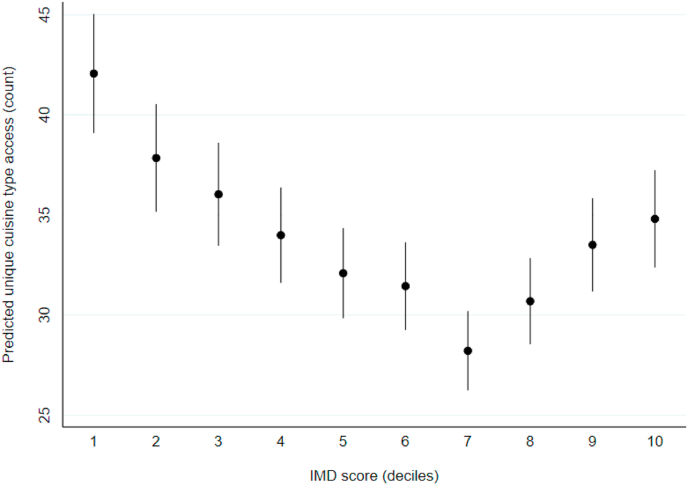
Number of unique cuisine types available online across postcode districts in England (n = 2088). Data points are predicted means with 95% CIs, calculated from IRRs estimated using negative binomial regression, controlled for postcode district rural urban classification, population density, the number of food outlets within the postcode district, and the number of food outlets accessible online.

In our controlled model, there was an inverse association between the number of unique cuisine types accessible online and deprivation. However, the predicted means with 95% CIs estimated from IRRs in Fig. 4 show evidence of a curvilinear relationship. The least deprived postcode districts had access to the greatest number of unique cuisine types (n = 42.1; 95% CI: 39.1, 45.0).

### 3.5 Association between deprivation and the percentage of neighbourhood food outlets accessible online

**Fig. 5:**
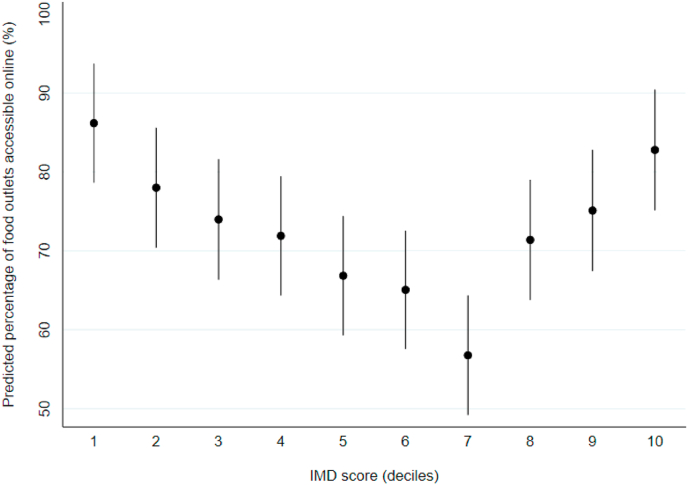
Number of food outlets accessible online as a percentage of the number physically accessible within the neighbourhood, across postcode districts in England (n = 2076). Data points are predicted means with 95% CIs, calculated from coefficients estimated using a general linear model, controlled for postcode district rural urban classification and population density.

In our controlled model, we observed evidence of a curvilinear relationship between deprivation and the number of food outlets accessible online expressed as a percentage of the number physically accessible within the neighbourhood. Predicted means with 95% CIs from coefficients are shown in Fig. 5. Postcode districts in deciles 2–9 of deprivation had a significantly lower percentage than postcode districts in decile 1. In the least deprived postcode districts, the number of food outlets accessible online as a percentage of the number of food outlets physically accessible within the neighbourhood was 86.2% (95% CI: 78.6, 93.7), which was greater than postcode districts in any other decile of deprivation.

### 3.6 Sensitivity analyses

In sensitivity analyses, we included five additional categories when determining the number of outlets within the boundaries of postcode districts (“*Cafes, snack bars and tea rooms*”, “*Convenience stores*”, “*Supermarkets*”, “*Bakeries*”, “*Delicatessens*”). The strength of associations were either similar or attenuated compared with our main analysis (appendix: tables A.6-A.9). The percentage of food outlets registered to accept orders online located within postcode districts continued to be positively associated with deprivation when additional food outlet types were included in the denominator. The number of food outlets and unique types of cuisine accessible online continued to be greatest in the most deprived postcode districts when we adjusted for additional food outlet types. Similarly, the curvilinear relationship between deprivation and the number of food outlets accessible online expressed as a percentage of the number physically accessible within the neighbourhood persisted.

## 4 Discussion

### 4.1 Summary of findings

To the best of our knowledge, we have described online food outlet access across a whole country for the first time in the international published literature. We found that in 2019, almost 30,000 food outlets in England were registered to accept orders online, which was around a third of the number of outlets that predominantly sell food prepared away-from-home. Per postcode district, a median of 64 food outlets and 39 unique cuisine types were accessible online. Online access was widespread and there was evidence that the number of food outlets accessible online was spatially patterned, and highest in urban regions of England. Moreover, the median number of food outlets accessible online, expressed as a percentage of the number physically accessible within the neighbourhood, was 63%. We observed evidence of socioeconomic patterning across our measures. Online food outlet access was greatest in the most deprived postcode districts in England. Additionally, the percentage of food outlets that predominantly sell food prepared away-from-home, registered to accept orders online, increased with deprivation. The number of unique cuisine types accessible online, and the number of food outlets accessible online expressed as a percentage of the number physically accessible within the neighbourhood were both greatest in the least deprived postcode districts. However, we observed evidence of a curvilinear relationship for these measures.

### 4.2 Interpretation of findings

The percentage of food outlets predominantly selling food prepared away-from-home located within postcode districts and registered to accept orders online increased with deprivation. In the most deprived postcode districts, the percentage of registered food outlets was around two times greater than in the least deprived postcode districts. The reasons for different levels of food outlet registration according to neighbourhood socioeconomic status have not been investigated to our knowledge. Some types of food outlet, including those typically registered to accept orders through online food delivery services, tend to cluster together in areas with greater deprivation (Ellaway et al., 2012; Maguire et al., 2015), perhaps due to lower rental costs for commercial premises, greater population density, or perceived demand (Mazidi & Speakman, 2017). In this context, registering to accept orders through an online food delivery service may be one way to ‘compete’ with other businesses and maximise potential custom. Although food outlets must pay initial registration fees and ongoing commission to online food delivery services (Li et al., 2020), it seems that even in more deprived areas, this does not outweigh the possible benefits. For example, the delivery areas of food outlets registered to accept orders online likely expands the customer catchment area, resulting in a larger potential customer base and a greater volume of orders. Overall there remains considerable scope for growth in the number of food outlets registered to accept orders online, including within the most deprived postcode districts. Future research that engages with food outlet owners to understand their rationale for registering to accept orders online is necessary to inform the case for, and development of, public health interventions.

Absolute online food outlet access was 50% greater in the most deprived postcode districts in England, compared to the least deprived, with some evidence of a dose-response association across the socioeconomic gradient. This online food outlet access may allow food to be ordered from outlets not normally accessible through other modes of order, resulting in changes in perceptions about food outlet accessibility, and how populations interact with their neighbourhood, both of which contribute to food purchasing decisions (Cervigni et al., 2020). In contrast to our finding, in one city in each of Australia, the Netherlands and USA, the number of food outlets accessible through an online food delivery service was not associated with area level socioeconomic status (Poelman et al., 2020). This previous research included 10 locations sampled from the most and least deprived areas of each city. We completed our study on a national scale and included all areas from across the socioeconomic gradient, helping to provide a more comprehensive assessment of online food outlet access.

It is not currently known if using an online food delivery service substitutes or supplements other ways of accessing food outlets and food prepared away-from-home. Having food outlet access across multiple modes of order within online and physical food environments could make the decision to purchase food prepared away-from-home easier and increase the frequency of this practice (Granheim et al., 2020). The number of food outlets accessible online compared to the number of food outlets physically accessible within the neighbourhood was similarly high in the least and most deprived postcode districts in England. If online food outlet access confers a health risk in addition to that posed by physical food outlet access, our finding suggests that groups of low and high socioeconomic status might be equally affected. However, absolute numbers of food outlets are typically greater in more deprived areas (Block et al., 2004; Maguire et al., 2015; Simon et al., 2008; Smoyer-Tomic et al., 2008). Since online food delivery services are an additional way that food prepared away-from-home can be purchased, online food outlet access compounds existing access through other modes of order. Moreover, food sold through these services is typically prepared in kitchen facilities of existing food outlets. This might mean that populations within postcode districts with the highest number of food outlets accessible online, which were often clustered together in the most deprived areas that typically have greatest neighbourhood access, may experience a ‘double-burden’ of disadvantage that exaggerates existing inequalities.

The number of unique cuisine types accessible online was inversely associated with deprivation and highest in the least deprived postcode districts. When registering to accept orders online, food outlets self-select the cuisine types used to categorise the food they sell. To gain a competitive advantage, food outlets may select a cuisine believed to differentiate themselves from others. However, we would have expected higher numbers of food outlets registered to accept orders online in more deprived areas to result in a reverse of the association we observed. Whilst the least deprived areas had access to the greatest number of unique cuisine types, they also had the lowest absolute online food outlet access. As a result, the number of food outlets available *within* each unique cuisine category would likely be lower than elsewhere. Whilst the number of unique cuisine types will contribute to meeting customer needs (Caspi et al., 2012), the role of having access to a greater number of food outlets *within* each unique cuisine category may also be important. Given that food purchasing decisions are influenced by multiple factors, including exposure to food marketing, for example (Janssen et al., 2018), knowledge about aspects of online food delivery services considered important by customers is needed to help further our understanding of this observation.

### 4.3 Public health implications and future research

As with physical access to food outlets that predominantly sell food prepared away-from-home, online food outlet access was greatest in the most deprived postcode districts of England. These modes of order coexist, which might contribute to the observed clustering of areas with the highest online food outlet access. In these areas in particular, overall greater access to food prepared away-from-home due to the opportunity to place orders online could be cause for public health concern. A greater number of food outlets accessible online could compound existing inequalities in diet and diet-related health since this exposure may be positively associated with frequency of online food delivery service use and consumption of food prepared away-from-home. Further research is required to understand the relationship between online food outlet *access* and online food delivery service *use*.

As of November 2019, across England, around one in three food outlets that predominantly sell food prepared away-from-home were registered to accept orders online. In the future, online food delivery services could become the primary way that food prepared away-from-home is purchased (Maimaiti et al., 2018). The COVID-19 pandemic (which our data precede) may have expedited this transition, as online food delivery service use is reported to have increased throughout this period (Leone et al., 2020). In part, this increased use could reflect a greater number of food outlets registering to accept orders online. In England, in response to the pandemic, urban planning regulations were relaxed to allow more food outlets to operate with a takeaway food function. It is feasible that food outlets subsequently registered to accept orders online to facilitate this (Chang et al., 2020). If food outlets realised a benefit to accepting orders online over this period of the pandemic, they may remain registered in the long term. Examining changes in the number of food outlets registered to accept orders online in the short- and long-term would help to quantify this potentially elevated public health risk. Moreover, this could inform future analysis that was outside of the scope of the current work. Further analyses for example, that aim to identify the extent to which the number of food outlets accessible online cluster together, and where, is warranted.

Public health actions directed towards online delivery services are not currently in place, but might be deemed necessary in the future (Bates et al., 2020). Such interventions would be implemented at the online food delivery service level yet apply to all registered food outlets, allowing widespread implementation. For example, food outlets in England are not required by law to display their food hygiene ratings inside their outlet (Food Standards Agency, 2018). Nonetheless, this information is provided for each food outlet registered to accept orders through the online food delivery service. In this case, a single entity (the online food delivery service) has applied an intervention that ensures uniform implementation at scale. A similar approach could be used for public health interventions. Online food delivery services have the potential to increase food outlet access to those with limited mobility or those in rural areas, which might benefit public health. However, the foods available online through these services are typically unhealthy (Partridge et al., 2020; Wang et al., 2021), therefore, greater access could be an additional public health burden. Lastly, public health interventions have been developed and adopted to address physical food outlet access through urban planning (zoning). For example, where new food outlets are not allowed to open in ‘exclusion zones’ (Keeble et al., 2019). However, the delivery areas of food outlets registered to accept orders online are not limited by administrative boundaries and do not respect the implementation of these regulations, meaning that they can deliver to areas where new outlets are not allowed to open. Moreover, a food outlet can be located in one postcode district not normally accessible in person, but be accessible online. This expanded food outlet access could threaten the effectiveness of existing place-based public health interventions.

### 4.4 Methodological considerations and limitations

As we understand it, this is the first study in the international published literature to investigate online food outlet access on a national scale. Nonetheless, our study is not without limitations. We generated a novel dataset through an automated data collection approach. If food outlets were registered to accept orders through the online food delivery service but not returned in our searches, it is possible that the data we collected were incomplete. However, according to annual reports published by the data source, around 30,000 food outlets were registered to accept orders at the time of data collection (Just Eat, 2019). This number was similar to the number we identified, increasing our confidence in the completeness of our data.

We used postcode districts as our unit of analysis. As such, our analyses may be subject to the modifiable areal unit problem (MAUP), whereby the spatial unit adopted for analyses has the potential to introduce bias (Fotheringham & Wong, 1991). However, postcode districts are employed by the online delivery service to help food outlets delineate their delivery areas, which justifies their use. Moreover, the MAUP is not unique to our study (Wilkins et al., 2019). Using postcode districts as our unit of analysis also meant that we were limited to using boundary data from 2012, which is subject to change over time. However, the conclusions drawn in our study are based on contemporaneous exposure and outcome data collected in 2019.

We conceptualised neighbourhood food environments as 1600m buffers around the geographic centroid of postcode districts. Previously, buffers ranging from 400m to 3200m have also been operationalised as ‘neighbourhoods’ (Wilkins et al., 2019). Our use of this buffer size may have influenced the magnitude of physical food outlet access within the neighbourhood. However, 1600m buffers have been shown to reflect the spatial extent of an individual's typical shopping behaviour, and this distance could be reasonably walked by an adult in around 15-20 minutes (Smith et al., 2010).

## 5 Conclusions

Our study is the first to investigate food outlet access through an online food delivery service on a national scale. Around one-third of food outlets that predominantly sell food prepared away-from-home were registered to accept orders online. Online food outlet access was greatest in the most deprived areas of England. As an alternative and complimentary mode of order, online food delivery services increase overall food outlet access. This increased food outlet access could drive more frequent purchasing of food prepared away-from-home and exaggerate existing health inequalities. Despite having lower online food outlet access, the number of unique cuisine types that were available was greatest in the least deprived areas, which could influence how frequently online food delivery services are used. Further research is needed to develop a better understanding of mechanisms underpinning the use of online food delivery services and how this practice subsequently affects dietary patterns and health.

## CRediT authorship contribution statement

**Matthew Keeble:** Conceptualization, Data curation, Formal analysis, Methodology, Writing – original draft, Writing – review & editing. **Jean Adams:** Conceptualization, Methodology, Supervision, Writing – review & editing. **Tom R.P. Bishop:** Data curation, Writing – review & editing. **Thomas Burgoine:** Conceptualization, Data curation, Formal analysis, Methodology, Supervision, Writing – review & editing.

## Declarations of competing interest

None.

## Figures and Tables

**Table A.1:** number of food outlets registered to accept orders online, and the number of food outlets located in the physical food environment of postcode districts in England (n = 2118).

	Number (total)
Online food delivery service ^a^	
Food outlets registered	29232
Physical food environment	
Food outlets within postcode district ^b^	82455
Food outlets within neighbourhood ^c^	376513

^a^ Data are counts, and from November 2019.

^b^ Food outlet categories included: Fast food and takeaway outlets; Fast food delivery services; Fish and Chip shops; Restaurants. Data from June 2019.

^c^ ‘Neighbourhood’ = 1600m Euclidean radius ‘neighbourhood’ buffer of postcode district geographic centroid.

**Table A.2:** Association between deprivation and the percentage of food outlets registered to accept online amongst postcode districts in England. Estimated using uncontrolled and controlled general linear models.

	Model 0^a^			Model 1^a^		
Percentage registered^b^ (%)	coef	95% CI		coef	95% CI	
IMD score (deciles)						
1 (4.28–10.21); least deprived	ref	–	–	ref	–	–
2 (10.22–12.08)	−0.96	−4.63	2.70	−0.84	−3.86	2.18
3 (12.09–14.00)	0.49	−3.16	4.13	1.27	−1.75	4.30
4 (14.01–15.91)	4.40	0.74	8.05	2.72	−0.30	5.74
5 (15.92–18.18)	3.92	0.26	7.58	2.29	−0.73	5.31
6 (18.19–20.60)	5.39	1.74	9.04	2.93	−0.09	5.95
7 (20.61–23.54)	8.10	4.44	11.75	4.18	1.14	7.22
8 (23.55–27.06)	16.15	12.49	19.80	9.75	6.68	12.82
9 (27.07–32.89)	21.80	18.14	25.45	11.12	8.01	14.23
10 (32.90–69.51); most deprived	29.03	25.37	32.69	20.02	16.93	23.10

^a^ Model 0 = uncontrolled. 2113 postcode districts included. Model 1 = controlled for postcode district population density and rural urban classification. 2084 postcode districts included.

^b^ Percentage registered = percentage of food outlets in postcode district registered to accept orders online.

**Table A.3:** Association between deprivation and online food outlet access amongst postcode districts in England. Estimated using uncontrolled and controlled negative binomial regression.

	Model 0^a^			Model 1^a^		
Accessible food outlets (count)	IRR^b^	95% CI		IRR^b^	95% CI	
IMD score (deciles)						
1 (4.28–10.21); least deprived	ref	–	–	ref	–	–
2 (10.22–12.08)	1.02	0.81	1.29	0.97	0.81	1.17
3 (12.09–14.00)	1.32	1.04	1.67	0.97	0.81	1.18
4 (14.01–15.91)	1.57	1.24	1.99	1.01	0.84	1.23
5 (15.92–18.18)	1.51	1.19	1.90	0.94	0.78	1.13
6 (18.19–20.60)	1.68	1.33	2.12	0.87	0.72	1.06
7 (20.61–23.54)	2.16	1.71	2.72	0.99	0.81	1.20
8 (23.55–27.06)	2.64	2.09	3.33	1.12	0.92	1.36
9 (27.07–32.89)	3.08	2.44	3.89	1.19	0.97	1.45
10 (32.90–69.51); most deprived	3.51	2.78	4.44	1.51	1.24	1.83

^a^ Model 0 = uncontrolled. 2118 postcode districts included. Model 1 = controlled for postcode district rural urban classification, population density, and the number of food outlets within their boundary. 2088 postcode districts included.

^b^ Incidence Rate Ratios (IRR) represent expected difference of outcome at each level of deprivation, compared to the reference group.

**Table A.4:** Association between deprivation and online unique cuisine type access amongst postcode districts in England. Estimated using uncontrolled and controlled negative binomial regression.

	Model 0^a^			Model 1^a^		
Unique cuisine types accessible (count)	IRR^b^	95% CI		IRR^b^	95% CI	
IMD score (deciles)						
1 (4.28–10.21); least deprived	ref	–	–	ref	–	–
2 (10.22–12.08)	0.96	0.80	1.14	0.90	0.82	0.99
3 (12.09–14.00)	1.05	0.88	1.25	0.86	0.78	0.95
4 (14.01–15.91)	1.18	1.00	1.41	0.81	0.73	0.89
5 (15.92–18.18)	1.12	0.94	1.33	0.76	0.69	0.84
6 (18.19–20.60)	1.21	1.02	1.44	0.75	0.68	0.83
7 (20.61–23.54)	1.39	1.17	1.66	0.67	0.61	0.74
8 (23.55–27.06)	1.62	1.37	1.93	0.73	0.66	0.81
9 (27.07–32.89)	1.87	1.58	2.23	0.80	0.72	0.88
10 (32.90–69.51); most deprived	2.08	1.75	2.48	0.83	0.75	0.92

^a^ Model 0 = uncontrolled. 2118 postcode districts included. Model 1 = controlled for postcode district rural urban classification, population density, the number of food outlets within their boundary and the number of food outlets accessible online. 2088 postcode districts included.

^b^ Incidence Rate Ratios (IRR) represent expected difference of outcome at each level of deprivation, compared to the reference group.

**Table A.5:** Association between deprivation and the percentage of neighbourhood food outlets accessible online amongst postcode districts in England. Estimated using uncontrolled and controlled general linear models.

	Model 0^a^			Model 1^a^		
Percentage accessible online^b^ (%)	coef	95% CI		coef	95% CI	
IMD score (deciles)						
1 (4.28–10.21); least deprived	ref	–	–	ref	–	–
2 (10.22–12.08)	−9.72	−20.63	1.20	−8.17	−18.80	2.46
3 (12.09–14.00)	−16.41	−27.28	−5.55	−12.19	−22.85	−1.53
4 (14.01–15.91)	−14.35	−25.21	−3.48	−14.28	−24.90	−3.65
5 (15.92–18.18)	−20.05	−30.92	−9.17	−19.32	−29.95	−8.69
6 (18.19–20.60)	−21.84	−32.67	−11.00	−21.11	−31.72	−10.51
7 (20.61–23.54)	−30.34	−41.21	−19.48	−29.40	−40.10	−18.69
8 (23.55–27.06)	−16.54	−27.41	−5.68	−14.79	−25.58	−3.99
9 (27.07–32.89)	−10.59	−21.43	0.25	−11.06	−21.97	−0.16
10 (32.90–69.51); most deprived	0.05	−10.80	10.90	−3.38	−14.22	7.45

^a^ Model 0 = uncontrolled. 2104 postcode districts included. Model 1 = controlled for postcode district population density and rural urban classification. 2076 postcode districts included.

^b^ Percentage accessible online = The number of food outlets accessible online as a percentage of the number physically accessible in the neighbourhood. ‘Neighbourhood’ = 1600m Euclidean radius ‘neighbourhood’ buffer of postcode district geographic centroid.

**Table A.6:** Sensitivity analyses: Association between deprivation and the percentage of food outlets registered to accept online amongst postcode districts in England. Estimated using uncontrolled and controlled general linear models and nine categories of food outlets from Ordnance Survey Points of Interest data ^a^.

	Model 0^b^			Model 1^b^		
Percentage registered^c^ (%)	coef	95% CI		coef	95% CI	
IMD score (deciles)						
1 (4.28–10.21); least deprived	ref	–	–	ref	–	–
2 (10.22–12.08)	−0.05	−1.83	1.72	0.03	−1.44	1.50
3 (12.09–14.00)	0.37	−1.40	2.15	0.77	−0.70	2.24
4 (14.01–15.91)	2.17	0.40	3.95	1.37	−0.10	2.84
5 (15.92–18.18)	2.59	0.82	4.37	1.83	0.36	3.30
6 (18.19–20.60)	2.51	0.74	4.29	1.35	−0.12	2.82
7 (20.61–23.54)	4.24	2.47	6.01	2.36	0.88	3.84
8 (23.55–27.06)	7.73	5.95	9.50	4.58	3.09	6.07
9 (27.07–32.89)	11.35	9.58	13.12	6.18	4.67	7.69
10 (32.90–69.51); most deprived	14.45	12.67	16.22	9.99	8.49	11.49

^a^ Food outlet categories included: Fast food and takeaway outlets, Fast food delivery services, Fish and Chip shops, Restaurants, Cafes, snack bars and tea rooms, Convenience stores, Supermarkets, Bakeries, Delicatessens.

^b^ Model 0 = uncontrolled. 2118 postcode districts included. Model 1 = controlled for postcode district population density and rural urban classification. 2088 postcode districts included.

^c^ Percentage registered = percentage of food outlets in postcode district registered to accept orders online.

**Table A.7:** Sensitivity analyses: Association between deprivation and online food outlet access amongst postcode districts in England. Estimated using uncontrolled and controlled negative binomial regression and nine categories of food outlets from Ordnance Survey Points of Interest data ^a^.

	Model 0^b^			Model 1^b^		
Accessible food outlets (count)	IRR^c^	95% CI		IRR^c^	95% CI	
IMD score (deciles)						
1 (4.28–10.21); least deprived	ref	–	–	ref	–	–
2 (10.22–12.08)	1.02	0.81	1.29	0.98	0.81	1.18
3 (12.09–14.00)	1.32	1.04	1.67	0.98	0.81	1.18
4 (14.01–15.91)	1.57	1.24	1.99	1.01	0.84	1.22
5 (15.92–18.18)	1.51	1.19	1.90	0.94	0.78	1.14
6 (18.19–20.60)	1.68	1.33	2.12	0.87	0.72	1.05
7 (20.61–23.54)	2.16	1.71	2.72	0.99	0.81	1.20
8 (23.55–27.06)	2.64	2.09	3.33	1.12	0.92	1.36
9 (27.07–32.89)	3.08	2.44	3.89	1.19	0.97	1.45
10 (32.90–69.51); most deprived	3.51	2.78	4.44	1.51	1.24	1.83

^a^ Food outlet categories included: Fast food and takeaway outlets, Fast food delivery services, Fish and Chip shops, Restaurants, Cafes, snack bars and tea rooms, Convenience stores, Supermarkets, Bakeries, Delicatessens.

^b^ Model 0 = uncontrolled. 2118 postcode districts included. Model 1 = controlled for postcode district population density and rural urban classification. 2087 postcode districts included.

^c^ Incidence Rate Ratios (IRR) represent expected difference of outcome at each level of deprivation, compared to the reference group.

**Table A.8:** Sensitivity analyses: Association between deprivation and online unique cuisine type access amongst postcode districts in England. Estimated using uncontrolled and controlled negative binomial regression and nine categories of food outlets from Ordnance Survey Points of Interest data ^a^.

	Model 0^b^			Model 1^b^		
Unique cuisine types accessible (count)	IRR^c^	95% CI		IRR^c^	95% CI	
IMD score (deciles)						
1 (4.28–10.21); least deprived	ref	–	–	ref	–	–
2 (10.22–12.08)	0.96	0.80	1.14	0.90	0.82	0.99
3 (12.09–14.00)	1.05	0.88	1.25	0.86	0.78	0.95
4 (14.01–15.91)	1.18	1.00	1.41	0.81	0.73	0.89
5 (15.92–18.18)	1.12	0.94	1.33	0.77	0.69	0.84
6 (18.19–20.60)	1.21	1.02	1.44	0.75	0.68	0.83
7 (20.61–23.54)	1.39	1.17	1.66	0.67	0.61	0.75
8 (23.55–27.06)	1.62	1.37	1.93	0.73	0.66	0.81
9 (27.07–32.89)	1.87	1.58	2.23	0.80	0.73	0.89
10 (32.90–69.51); most deprived	2.08	1.75	2.48	0.83	0.75	0.92

^a^ Food outlet categories included: Fast food and takeaway outlets, Fast food delivery services, Fish and Chip shops, Restaurants, Cafes, snack bars and tea rooms, Convenience stores, Supermarkets, Bakeries, Delicatessens.

^b^ Model 0 = uncontrolled. 2118 postcode districts included. Model 1 = controlled for postcode district rural urban classification, population density, the number of food outlets within their boundary and the number of food outlets accessible online. 2088 postcode districts included.

^c^ Incidence Rate Ratios (IRR) represent expected difference of outcome at each level of deprivation, compared to the reference group.

**Table A.9:** Sensitivity analyses: Association between deprivation and the percentage of neighbourhood food outlets accessible online amongst postcode districts in England. Estimated using uncontrolled and controlled general linear models and nine categories of food outlets from Ordnance Survey Points of Interest data ^a^.

	Model 0^b^			Model 1^b^		
Percentage accessible online^c^ (%)	β.	95% CI		β.	95% CI	
IMD score (deciles)						
1 (4.28–10.21); least deprived	ref	–	–	ref	–	–
2 (10.22–12.08)	−0.44	−7.09	6.22	0.59	−6.00	7.19
3 (12.09–14.00)	−2.08	−8.71	4.55	−0.09	−6.70	6.53
4 (14.01–15.91)	−6.66	−13.31	−0.01	−6.69	−13.30	−0.08
5 (15.92–18.18)	−7.07	−13.72	−0.42	−6.62	−13.22	−0.02
6 (18.19–20.60)	−9.49	−16.13	−2.85	−9.13	−15.73	−2.53
7 (20.61–23.54)	−12.40	−19.04	−5.75	−12.07	−18.72	−5.41
8 (23.55–27.06)	−6.08	−12.73	0.57	−5.38	−12.09	1.33
9 (27.07–32.89)	−1.99	−8.63	4.65	−2.31	−9.10	4.48
10 (32.90–69.51); most deprived	3.08	−3.57	9.73	1.18	−5.57	7.92

^a^ Food outlet categories included: Fast food and takeaway outlets, Fast food delivery services, Fish and Chip shops, Restaurants, Cafes, snack bars and tea rooms, Convenience stores, Supermarkets, Bakeries, Delicatessens.

^b^ Model 0 = uncontrolled. 2104 postcode districts included. Model 1 = controlled for postcode district population density and rural urban classification. 2087 postcode districts included.

^c^ Percentage accessible online = The number of food outlets accessible online as a percentage of the number physically accessible in the neighbourhood. ‘Neighbourhood’ = 1600m Euclidean radius ‘neighbourhood’ buffer of postcode district geographic centroid.
