# Supplementary material for: Socioeconomic inequalities in food outlet access through an online food delivery service in England: A cross-sectional descriptive analysis
Source: Appl Geogr. 2021 Aug;133:None. doi: 10.1016/j.apgeog.2021.102498 (PMC8288297; doi:10.1016/j.apgeog.2021.102498)
Supplement: Multimedia component 1 [file mmc1.docx]

| Table A.1: number of food outlets registered to accept orders online, and the number of food outlets located in the physical food environment of postcode districts in England (n=2118). | |
| --- | --- |
|  | Number (total) |
| **Online food delivery service** ^a^ |  |
| Food outlets registered | 29232 |
| **Physical food environment** |  |
| Food outlets within postcode district ^b^ | 82455 |
| Food outlets within neighbourhood ^c^ | 376513 |
| ^a^ Data are counts, and from November 2019.  ^b^ Food outlet categories included: Fast food and takeaway outlets; Fast food delivery services; Fish and Chip shops; Restaurants. Data from June 2019.  ^c^ ‘Neighbourhood’ = 1600m Euclidean radius ‘neighbourhood’ buffer of postcode district geographic centroid | |

| Table A.2: Association between deprivation and the percentage of food outlets registered to accept online amongst postcode districts in England. Estimated using uncontrolled and controlled general linear models. | | | | | | | |
| --- | --- | --- | --- | --- | --- | --- | --- |
|  | Model 0 ^a^ | | | Model 1 ^a^ | | |  |
| **Percentage registered** ^b^ (%) | coef | 95% CI | | coef | 95% CI | |  |
| IMD score (deciles) |  |  |  |  |  |  |  |
| 1 (4.28-10.21); least deprived | ref | - | - | ref | - | - |  |
| 2 (10.22-12.08) | -0.96 | -4.63 | 2.70 | -0.84 | -3.86 | 2.18 |  |
| 3 (12.09-14.00) | 0.49 | -3.16 | 4.13 | 1.27 | -1.75 | 4.30 |  |
| 4 (14.01-15.91) | 4.40 | 0.74 | 8.05 | 2.72 | -0.30 | 5.74 |  |
| 5 (15.92-18.18) | 3.92 | 0.26 | 7.58 | 2.29 | -0.73 | 5.31 |  |
| 6 (18.19-20.60) | 5.39 | 1.74 | 9.04 | 2.93 | -0.09 | 5.95 |  |
| 7 (20.61-23.54) | 8.10 | 4.44 | 11.75 | 4.18 | 1.14 | 7.22 |  |
| 8 (23.55-27.06) | 16.15 | 12.49 | 19.80 | 9.75 | 6.68 | 12.82 |  |
| 9 (27.07-32.89) | 21.80 | 18.14 | 25.45 | 11.12 | 8.01 | 14.23 |  |
| 10 (32.90-69.51); most deprived | 29.03 | 25.37 | 32.69 | 20.02 | 16.93 | 23.10 |  |
| ^a^ Model 0 = uncontrolled. 2113 postcode districts included. Model 1 = controlled for postcode district population density and rural urban classification. 2084 postcode districts included.  ^b^ Percentage registered = percentage of food outlets in postcode district registered to accept orders online. | | | | | | |  |

| Table A.3: Association between deprivation and online food outlet access amongst postcode districts in England. Estimated using uncontrolled and controlled negative binomial regression. | | | | | | | |
| --- | --- | --- | --- | --- | --- | --- | --- |
|  | Model 0 ^a^ | | | Model 1 ^a^ | | |  |
| **Accessible food outlets** (count) | IRR ^b^ | 95% CI | | IRR ^b^ | 95% CI | |  |
| IMD score (deciles) |  |  |  |  |  |  |  |
| 1 (4.28-10.21); least deprived | ref | - | - | ref | - | - |  |
| 2 (10.22-12.08) | 1.02 | 0.81 | 1.29 | 0.97 | 0.81 | 1.17 |  |
| 3 (12.09-14.00) | 1.32 | 1.04 | 1.67 | 0.97 | 0.81 | 1.18 |  |
| 4 (14.01-15.91) | 1.57 | 1.24 | 1.99 | 1.01 | 0.84 | 1.23 |  |
| 5 (15.92-18.18) | 1.51 | 1.19 | 1.90 | 0.94 | 0.78 | 1.13 |  |
| 6 (18.19-20.60) | 1.68 | 1.33 | 2.12 | 0.87 | 0.72 | 1.06 |  |
| 7 (20.61-23.54) | 2.16 | 1.71 | 2.72 | 0.99 | 0.81 | 1.20 |  |
| 8 (23.55-27.06) | 2.64 | 2.09 | 3.33 | 1.12 | 0.92 | 1.36 |  |
| 9 (27.07-32.89) | 3.08 | 2.44 | 3.89 | 1.19 | 0.97 | 1.45 |  |
| 10 (32.90-69.51); most deprived | 3.51 | 2.78 | 4.44 | 1.51 | 1.24 | 1.83 |  |
| ^a^ Model 0 = uncontrolled. 2118 postcode districts included. Model 1 = controlled for postcode district rural urban classification, population density, and the number of food outlets within their boundary. 2088 postcode districts included.  ^b^ Incidence Rate Ratios (IRR) represent expected difference of outcome at each level of deprivation, compared to the reference group. | | | | | | |  |

| Table A.4: Association between deprivation and online unique cuisine type access amongst postcode districts in England. Estimated using uncontrolled and controlled negative binomial regression. | | | | | | | |
| --- | --- | --- | --- | --- | --- | --- | --- |
|  | Model 0 ^a^ | | | Model 1 ^a^ | | |  |
| **Unique cuisine types accessible** (count) | IRR ^b^ | 95% CI | | IRR ^b^ | 95% CI | |  |
| IMD score (deciles) |  |  |  |  |  |  |  |
| 1 (4.28-10.21); least deprived | ref | - | - | ref | - | - |  |
| 2 (10.22-12.08) | 0.96 | 0.80 | 1.14 | 0.90 | 0.82 | 0.99 |  |
| 3 (12.09-14.00) | 1.05 | 0.88 | 1.25 | 0.86 | 0.78 | 0.95 |  |
| 4 (14.01-15.91) | 1.18 | 1.00 | 1.41 | 0.81 | 0.73 | 0.89 |  |
| 5 (15.92-18.18) | 1.12 | 0.94 | 1.33 | 0.76 | 0.69 | 0.84 |  |
| 6 (18.19-20.60) | 1.21 | 1.02 | 1.44 | 0.75 | 0.68 | 0.83 |  |
| 7 (20.61-23.54) | 1.39 | 1.17 | 1.66 | 0.67 | 0.61 | 0.74 |  |
| 8 (23.55-27.06) | 1.62 | 1.37 | 1.93 | 0.73 | 0.66 | 0.81 |  |
| 9 (27.07-32.89) | 1.87 | 1.58 | 2.23 | 0.80 | 0.72 | 0.88 |  |
| 10 (32.90-69.51); most deprived | 2.08 | 1.75 | 2.48 | 0.83 | 0.75 | 0.92 |  |
| ^a^ Model 0 = uncontrolled. 2118 postcode districts included. Model 1 = controlled for postcode district rural urban classification, population density, the number of food outlets within their boundary and the number of food outlets accessible online. 2088 postcode districts included.  ^b^ Incidence Rate Ratios (IRR) represent expected difference of outcome at each level of deprivation, compared to the reference group. | | | | | | |  |

| Table A.5: Association between deprivation and the percentage of neighbourhood food outlets accessible online amongst postcode districts in England. Estimated using uncontrolled and controlled general linear models. | | | | | | | |
| --- | --- | --- | --- | --- | --- | --- | --- |
|  | Model 0 ^a^ | | | Model 1 ^a^ | | |  |
| **Percentage accessible online** ^b^ (%) | coef | 95% CI | | coef | 95% CI | |  |
| IMD score (deciles) |  |  |  |  |  |  |  |
| 1 (4.28-10.21); least deprived | ref | - | - | ref | - | - |  |
| 2 (10.22-12.08) | -9.72 | -20.63 | 1.20 | -8.17 | -18.80 | 2.46 |  |
| 3 (12.09-14.00) | -16.41 | -27.28 | -5.55 | -12.19 | -22.85 | -1.53 |  |
| 4 (14.01-15.91) | -14.35 | -25.21 | -3.48 | -14.28 | -24.90 | -3.65 |  |
| 5 (15.92-18.18) | -20.05 | -30.92 | -9.17 | -19.32 | -29.95 | -8.69 |  |
| 6 (18.19-20.60) | -21.84 | -32.67 | -11.00 | -21.11 | -31.72 | -10.51 |  |
| 7 (20.61-23.54) | -30.34 | -41.21 | -19.48 | -29.40 | -40.10 | -18.69 |  |
| 8 (23.55-27.06) | -16.54 | -27.41 | -5.68 | -14.79 | -25.58 | -3.99 |  |
| 9 (27.07-32.89) | -10.59 | -21.43 | 0.25 | -11.06 | -21.97 | -0.16 |  |
| 10 (32.90-69.51); most deprived | 0.05 | -10.80 | 10.90 | -3.38 | -14.22 | 7.45 |  |
| ^a^ Model 0 = uncontrolled. 2104 postcode districts included. Model 1 = controlled for postcode district population density and rural urban classification. 2076 postcode districts included.  ^b^ Percentage accessible online = The number of food outlets accessible online as a percentage of the number physically accessible in the neighbourhood. ‘Neighbourhood’ = 1600m Euclidean radius ‘neighbourhood’ buffer of postcode district geographic centroid. | | | | | | |  |

| Table A.6: Sensitivity analyses: Association between deprivation and the percentage of food outlets registered to accept online amongst postcode districts in England. Estimated using uncontrolled and controlled general linear models and nine categories of food outlets from Ordnance Survey Points of Interest data ^a^. | | | | | | | |
| --- | --- | --- | --- | --- | --- | --- | --- |
|  | Model 0 ^b^ | | | Model 1 ^b^ | | |  |
| **Percentage registered** ^c^ (%) | coef | 95% CI | | coef | 95% CI | |  |
| IMD score (deciles) |  |  |  |  |  |  |  |
| 1 (4.28-10.21); least deprived | ref | - | - | ref | - | - |  |
| 2 (10.22-12.08) | -0.05 | -1.83 | 1.72 | 0.03 | -1.44 | 1.50 |  |
| 3 (12.09-14.00) | 0.37 | -1.40 | 2.15 | 0.77 | -0.70 | 2.24 |  |
| 4 (14.01-15.91) | 2.17 | 0.40 | 3.95 | 1.37 | -0.10 | 2.84 |  |
| 5 (15.92-18.18) | 2.59 | 0.82 | 4.37 | 1.83 | 0.36 | 3.30 |  |
| 6 (18.19-20.60) | 2.51 | 0.74 | 4.29 | 1.35 | -0.12 | 2.82 |  |
| 7 (20.61-23.54) | 4.24 | 2.47 | 6.01 | 2.36 | 0.88 | 3.84 |  |
| 8 (23.55-27.06) | 7.73 | 5.95 | 9.50 | 4.58 | 3.09 | 6.07 |  |
| 9 (27.07-32.89) | 11.35 | 9.58 | 13.12 | 6.18 | 4.67 | 7.69 |  |
| 10 (32.90-69.51); most deprived | 14.45 | 12.67 | 16.22 | 9.99 | 8.49 | 11.49 |  |
| ^a^ Food outlet categories included: Fast food and takeaway outlets, Fast food delivery services, Fish and Chip shops, Restaurants, Cafes, snack bars and tea rooms, Convenience stores, Supermarkets, Bakeries, Delicatessens.  ^b^ Model 0 = uncontrolled. 2118 postcode districts included. Model 1 = controlled for postcode district population density and rural urban classification. 2088 postcode districts included.  ^c^ Percentage registered = percentage of food outlets in postcode district registered to accept orders online. | | | | | | |  |

| Table A.7: Sensitivity analyses: Association between deprivation and online food outlet access amongst postcode districts in England. Estimated using uncontrolled and controlled negative binomial regression and nine categories of food outlets from Ordnance Survey Points of Interest data ^a^. | | | | | | | |
| --- | --- | --- | --- | --- | --- | --- | --- |
|  | Model 0 ^b^ | | | Model 1 ^b^ | | |  |
| **Accessible food outlets** (count) | IRR ^c^ | 95% CI | | IRR ^c^ | 95% CI | |  |
| IMD score (deciles) |  |  |  |  |  |  |  |
| 1 (4.28-10.21); least deprived | ref | - | - | ref | - | - |  |
| 2 (10.22-12.08) | 1.02 | 0.81 | 1.29 | 0.98 | 0.81 | 1.18 |  |
| 3 (12.09-14.00) | 1.32 | 1.04 | 1.67 | 0.98 | 0.81 | 1.18 |  |
| 4 (14.01-15.91) | 1.57 | 1.24 | 1.99 | 1.01 | 0.84 | 1.22 |  |
| 5 (15.92-18.18) | 1.51 | 1.19 | 1.90 | 0.94 | 0.78 | 1.14 |  |
| 6 (18.19-20.60) | 1.68 | 1.33 | 2.12 | 0.87 | 0.72 | 1.05 |  |
| 7 (20.61-23.54) | 2.16 | 1.71 | 2.72 | 0.99 | 0.81 | 1.20 |  |
| 8 (23.55-27.06) | 2.64 | 2.09 | 3.33 | 1.12 | 0.92 | 1.36 |  |
| 9 (27.07-32.89) | 3.08 | 2.44 | 3.89 | 1.19 | 0.97 | 1.45 |  |
| 10 (32.90-69.51); most deprived | 3.51 | 2.78 | 4.44 | 1.51 | 1.24 | 1.83 |  |
| ^a^ Food outlet categories included: Fast food and takeaway outlets, Fast food delivery services, Fish and Chip shops, Restaurants, Cafes, snack bars and tea rooms, Convenience stores, Supermarkets, Bakeries, Delicatessens.  ^b^ Model 0 = uncontrolled. 2118 postcode districts included. Model 1 = controlled for postcode district population density and rural urban classification. 2087 postcode districts included.  ^c^ Incidence Rate Ratios (IRR) represent expected difference of outcome at each level of deprivation, compared to the reference group. | | | | | | |  |

| Table A.8: Sensitivity analyses: Association between deprivation and online unique cuisine type access amongst postcode districts in England. Estimated using uncontrolled and controlled negative binomial regression and nine categories of food outlets from Ordnance Survey Points of Interest data ^a^. | | | | | | | |
| --- | --- | --- | --- | --- | --- | --- | --- |
|  | Model 0 ^b^ | | | Model 1 ^b^ | | |  |
| **Unique cuisine types accessible** (count) | IRR ^c^ | 95% CI | | IRR ^c^ | 95% CI | |  |
| IMD score (deciles) |  |  |  |  |  |  |  |
| 1 (4.28-10.21); least deprived | ref | - | - | ref | - | - |  |
| 2 (10.22-12.08) | 0.96 | 0.80 | 1.14 | 0.90 | 0.82 | 0.99 |  |
| 3 (12.09-14.00) | 1.05 | 0.88 | 1.25 | 0.86 | 0.78 | 0.95 |  |
| 4 (14.01-15.91) | 1.18 | 1.00 | 1.41 | 0.81 | 0.73 | 0.89 |  |
| 5 (15.92-18.18) | 1.12 | 0.94 | 1.33 | 0.77 | 0.69 | 0.84 |  |
| 6 (18.19-20.60) | 1.21 | 1.02 | 1.44 | 0.75 | 0.68 | 0.83 |  |
| 7 (20.61-23.54) | 1.39 | 1.17 | 1.66 | 0.67 | 0.61 | 0.75 |  |
| 8 (23.55-27.06) | 1.62 | 1.37 | 1.93 | 0.73 | 0.66 | 0.81 |  |
| 9 (27.07-32.89) | 1.87 | 1.58 | 2.23 | 0.80 | 0.73 | 0.89 |  |
| 10 (32.90-69.51); most deprived | 2.08 | 1.75 | 2.48 | 0.83 | 0.75 | 0.92 |  |
| ^a^ Food outlet categories included: Fast food and takeaway outlets, Fast food delivery services, Fish and Chip shops, Restaurants, Cafes, snack bars and tea rooms, Convenience stores, Supermarkets, Bakeries, Delicatessens.  ^b^ Model 0 = uncontrolled. 2118 postcode districts included. Model 1 = controlled for postcode district rural urban classification, population density, the number of food outlets within their boundary and the number of food outlets accessible online. 2088 postcode districts included.  ^c^ Incidence Rate Ratios (IRR) represent expected difference of outcome at each level of deprivation, compared to the reference group. | | | | | | |  |

| Table A.9: Sensitivity analyses: Association between deprivation and the percentage of neighbourhood food outlets accessible online amongst postcode districts in England. Estimated using uncontrolled and controlled general linear models and nine categories of food outlets from Ordnance Survey Points of Interest data ^a^. | | | | | | | |
| --- | --- | --- | --- | --- | --- | --- | --- |
|  | Model 0 ^b^ | | | Model 1 ^b^ | | |  |
| **Percentage accessible online** ^c^ (%) | β. | 95% CI | | β. | 95% CI | |  |
| IMD score (deciles) |  |  |  |  |  |  |  |
| 1 (4.28-10.21); least deprived | ref | - | - | ref | - | - |  |
| 2 (10.22-12.08) | -0.44 | -7.09 | 6.22 | 0.59 | -6.00 | 7.19 |  |
| 3 (12.09-14.00) | -2.08 | -8.71 | 4.55 | -0.09 | -6.70 | 6.53 |  |
| 4 (14.01-15.91) | -6.66 | -13.31 | -0.01 | -6.69 | -13.30 | -0.08 |  |
| 5 (15.92-18.18) | -7.07 | -13.72 | -0.42 | -6.62 | -13.22 | -0.02 |  |
| 6 (18.19-20.60) | -9.49 | -16.13 | -2.85 | -9.13 | -15.73 | -2.53 |  |
| 7 (20.61-23.54) | -12.40 | -19.04 | -5.75 | -12.07 | -18.72 | -5.41 |  |
| 8 (23.55-27.06) | -6.08 | -12.73 | 0.57 | -5.38 | -12.09 | 1.33 |  |
| 9 (27.07-32.89) | -1.99 | -8.63 | 4.65 | -2.31 | -9.10 | 4.48 |  |
| 10 (32.90-69.51); most deprived | 3.08 | -3.57 | 9.73 | 1.18 | -5.57 | 7.92 |  |
| ^a^ Food outlet categories included: Fast food and takeaway outlets, Fast food delivery services, Fish and Chip shops, Restaurants, Cafes, snack bars and tea rooms, Convenience stores, Supermarkets, Bakeries, Delicatessens.  ^b^ Model 0 = uncontrolled. 2104 postcode districts included. Model 1 = controlled for postcode district population density and rural urban classification. 2087 postcode districts included.  ^c^ Percentage accessible online = The number of food outlets accessible online as a percentage of the number physically accessible in the neighbourhood. ‘Neighbourhood’ = 1600m Euclidean radius ‘neighbourhood’ buffer of postcode district geographic centroid. | | | | | | |  |
