# Supplementary material for: Socioeconomic inequalities in food outlet access through an online food delivery service in England: A cross-sectional descriptive analysis
Source: Appl Geogr. 2021 Aug;133:None. doi: 10.1016/j.apgeog.2021.102498 (PMC8288297; doi:10.1016/j.apgeog.2021.102498)
Supplement: Multimedia component 2 [file mmc2.pdf]

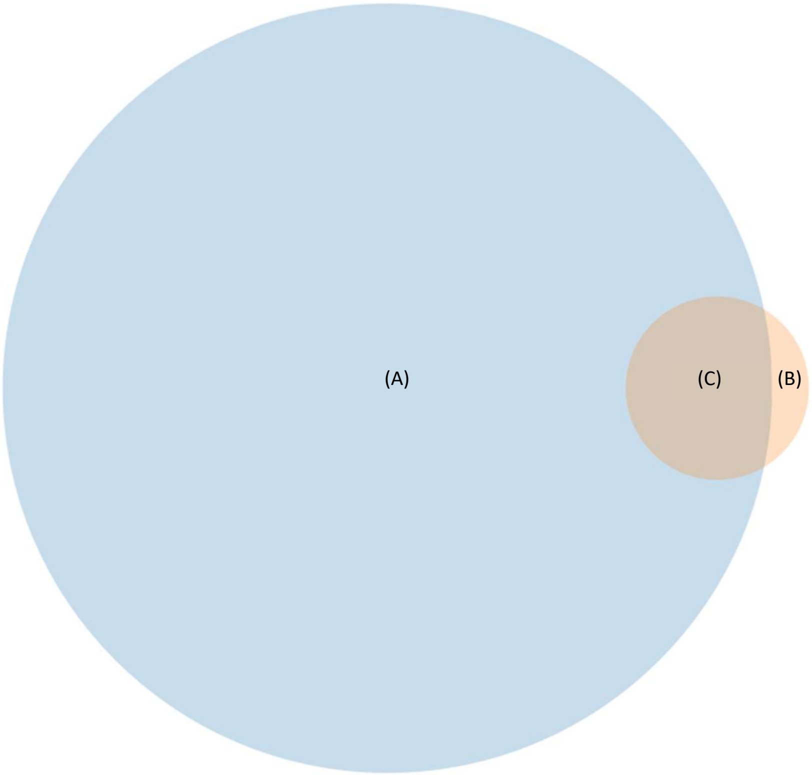

Supplementary material: Comparison between food outlets registered with Just Eat and Deliveroo in one region in England. Data are from April 2020.

(A) = food outlets registered to accept orders through Just Eat. N = **1673**
(B) = Food outlets registered to accept orders through Deliveroo. N = **334**
(C) = Food outlets exclusively registered to accept orders through Deliveroo. N = **91**
